# Estimated pulse wave velocity improves risk stratification for all-cause mortality in patients with COVID-19

**DOI:** 10.1038/s41598-021-99050-0

**Published:** 2021-10-12

**Authors:** Kimon Stamatelopoulos, Georgios Georgiopoulos, Kenneth F. Baker, Giusy Tiseo, Dimitrios Delialis, Charalampos Lazaridis, Greta Barbieri, Stefano Masi, Nikolaos I. Vlachogiannis, Kateryna Sopova, Alessandro Mengozzi, Lorenzo Ghiadoni, Ina Schim van der Loeff, Aidan T. Hanrath, Bajram Ajdini, Charalambos Vlachopoulos, Meletios A. Dimopoulos, Christopher J. A. Duncan, Marco Falcone, Konstantinos Stellos, the Pisa COVID-19 Research Group, Giusy Tiseo, Greta Barbieri, Stefano Masi, Alessandro Mengozzi, Lorenzo Ghiadoni, Marco Falcone, Fabio Monzani, Francesco Menichetti, Agostino Virdis, Francesco Forfori, Baldassarri Rubia, Bertini Pietro, Brizzi Giulia, Corradi Francesco, Della Rocca Alessandra, Guarracino Fabio, Malacarne Paolo, Monfroni Marco, Piagnani Chiara, Park Naria, Celi Alessandro, Laura Carrozzi, Cinotti Francesco, Massimo Santini, Cipriano Alessandro, Biancalana Martina, Borselli Matteo, Nencini Elia, Spinelli Stefano, Ruberti Francesca, Forotti Giovanna, Sciuto Maria, Salvatore De Marco, Antognoli Rachele, Calsolario Valeria, Paterni Simone, Colangelo Luciano, Sonato Chiara, Galfo Valentina, Monica Uliana, Newcastle COVID-19 Research Group, Kenneth F. Baker, Ina Schim van der Loeff, Aidan T. Hanrath, Christopher J. A. Duncan, Su Ann Tee, Richard Capstick, Gabriella Marchitelli, Ang Li, Andrew Barr, Alsafi Eid, Sajeel Ahmed, Dalvir Bajwa, Omer Mohammed

**Affiliations:** 1 Department of Clinical Therapeutics, National and Kapodistrian University of Athens School of Medicine, Athens, Greece; 2 Biosciences Institute, International Centre for Life, Vascular Biology and Medicine Theme, Faculty of Medical Sciences, Newcastle University, Central Parkway, Newcastle upon Tyne, NE1 3BZ UK; 3 School of Biomedical Engineering and Imaging Sciences, King’s College, London, UK; 4 Translational and Clinical Research Institute, Newcastle University, Newcastle upon Tyne, UK; 5 NIHR Newcastle Biomedical Research Centre, Newcastle University and Newcastle upon Tyne Hospitals NHS Foundation Trust, Newcastle upon Tyne, UK; 6 Department of Clinical and Experimental Medicine, University of Pisa, Pisa, Italy; 7 RVI and Freeman Hospitals, Newcastle upon Tyne Hospitals NHS Foundation Trust, Newcastle upon Tyne, UK; 8 First Department of Cardiology, National and Kapodistrian University of Athens Medical School, Athens, Greece; 9 Department of Anaesthesia and Intensive Care, University Hospital of Pisa, Pisa, Italy; 10 Department of Cardiothoracic and Vascular Department, University of Pisa, Pisa, Italy; 11 Department of Emergency Medicine, Azienda Ospedaliera Universitaria Pisana, Pisa, Italy; 12 Fifth Medical Unit, Azienda Ospedaliera Universitaria Pisana, Pisa, Italy; 13 Geriatrics Unit, Azienda Ospedaliera Universitaria Pisana, Pisa, Italy; 14 Infectious Diseases Unit, Azienda Ospedaliera Universitaria Pisana, Pisa, Italy; 15 Fourth Medical Unit, Azienda Ospedaliera Universitaria Pisana, Pisa, Italy

**Keywords:** Viral infection, Predictive markers, Prognostic markers

## Abstract

Accurate risk stratification in COVID-19 patients consists a major clinical need to guide therapeutic strategies. We sought to evaluate the prognostic role of estimated pulse wave velocity (ePWV), a marker of arterial stiffness which reflects overall arterial integrity and aging, in risk stratification of hospitalized patients with COVID-19. This retrospective, longitudinal cohort study, analyzed a total population of 1671 subjects consisting of 737 hospitalized COVID-19 patients consecutively recruited from two tertiary centers (Newcastle cohort: n = 471 and Pisa cohort: n = 266) and a non-COVID control cohort (n = 934). Arterial stiffness was calculated using validated formulae for ePWV. ePWV progressively increased across the control group, COVID-19 survivors and deceased patients (adjusted mean increase per group 1.89 m/s, *P* < 0.001). Using a machine learning approach, ePWV provided incremental prognostic value and improved reclassification for mortality over the core model including age, sex and comorbidities [AUC (core model + ePWV vs. core model) = 0.864 vs. 0.755]. ePWV provided similar prognostic value when pulse pressure or hs-Troponin were added to the core model or over its components including age and mean blood pressure (*p* < 0.05 for all). The optimal prognostic ePWV value was 13.0 m/s. ePWV conferred additive discrimination (AUC: 0.817 versus 0.779, *P* < 0.001) and reclassification value (NRI = 0.381, *P* < 0.001) over the 4C Mortality score, a validated score for predicting mortality in COVID-19 and the Charlson comorbidity index. We suggest that calculation of ePWV, a readily applicable estimation of arterial stiffness, may serve as an additional clinical tool to refine risk stratification of hospitalized patients with COVID-19 beyond established risk factors and scores.

## Introduction

The coronavirus disease 2019 (COVID-19), caused by severe acute respiratory coronavirus 2 (SARS-CoV-2) infection, was declared a global pandemic in March 2020 by the World Health Organization. Due to the need for a swift triage of therapeutic interventions in acute COVID-19, accurate risk stratification at admission of these patients is of clinical importance. Epidemiological data regarding the connection between COVID-19 and the cardiovascular system point out that hypertension is one of the most common comorbidities in COVID-19^1^ and a determinant of all-cause mortality in hospitalized COVID-19 patients^2,3^. Moreover, age and cardiovascular disease (CVD)^4,5^ have also emerged as independent risk factors for worse outcomes in COVID-19. Aging and arterial blood pressure (BP) levels are considered the two major components of arterial stiffness, an independent predictor of cardiovascular events and a marker of vascular aging and hypertension-mediated organ damage (HMOD)^6^, severity of hypertension and arterial integrity^7,8^. Thus, it is tempting to hypothesize that increased arterial stiffness may provide additive prognostic information in COVID-19 patients. The gold-standard non-invasive method for measuring large artery stiffness is carotid-femoral pulse wave velocity (cfPWV)^6^. However, due to specialized equipment required for measuring cfPWV, it is not practical to be routinely assessed in a pandemic setting. Any additional equipment would increase the contact time between care-giver and patient increasing risk of transmission. Alternatively, an estimated measure of PWV (ePWV), using age and BP levels, has been developed^9^ in non-COVID-19 European populations with additive prognostic value for future CV events over traditional risk factors and scoring systems^10,11^. The clinical value of arterial stiffness, as calculated by ePWV, in predicting the outcome of COVID-19 remains unknown. To address this gap in knowledge, we assessed the additive prognostic value of ePWV for mortality of hospitalized COVID-19 patients. We also compared ePWV performance against other readily available BP markers associated with outcome in COVID-19 patients, as well as in a control non-COVID-19 cohort.

## Results

### Population characteristics

Table 1 summarizes the descriptive characteristics of the combined Newcastle and Pisa cohorts. During the first 28 days after diagnosis, 184 (24.97%) patients died. Importantly, mortality rate was similar between months of admission (Table S2). Among other parameters known to be associated with increased mortality in COVID-19 patients, ePWV was higher in deceased patients (Table 1). Comparison of the two COVID-19 cohorts is depicted in Table S3.

**Table 1:** Descriptive characteristics of the combined Newcastle and Pisa cohorts of hospitalised patients with COVID-19.

	All	Survived	Deceased	*P* value
		553 (75.03)	184 (24.97)	
Age (years)*	72(58–83)	67(55–79)	82(76–87)	< 0.001
Male sex	309 (41.9)	242 (43.8)	67 (36.4)	0.08
CKD	136 (18.5)	83 (15.1)	53 (28.8)	< 0.001
CAD	181 (24.6)	113 (20.4)	58 (31.5)	0.002
Heart failure	93 (12.6)	44 (7.9)	35 (19.0)	< 0.001
Smoking	28 (4.3)	23 (4.7)	5 (3.1)	0.372
Hyperlipidemia	44 (6.0)	30 (5.4)	14 (7.6)	0.279
Diabetes mellitus	175 (23.7)	123 (22.2)	52 (28.3)	0.097
Hypertension	311 (42.3)	205 (37.21)	106 (57.6)	< 0.001
Cancer	95(12.9)	65 (11.75)	30 (16.3)	0.111
Lung disease	169 (22.9)	121 (21.0)	48 (26.1)	0.24
SBP (mmHg)	129 (22.5)	129 (22.0)	127 (24.3)	0.247
DBP (mmHg)	73.7 (14.0)	74.4 (13.6)	71.6 (15.2)	0.02
PP (mmHg)	54.9 (19.2)	54.7 (18.2)	55.3 (21.8)	0.733
MBP (mmHg)	95.6 (15.3)	96.3 (14.9)	93.7 (16.2)	0.049
Days of hospitalisation*	10 (5–19)	11.5(6–21)	7(4–13)	< 0.001
WBC count (10^3^/ml)*	7.16 (5.3–9.4)	7.1 (5.2–9.1)	7.47(5.6–10.7)	0.014
Lymphocyte count(/ml)*	940 (640–1330)	970 (680–1370)	810 (520–1201)	< 0.001
CRP (mg/L)*	68 (28–135)	59 (23–127)	89.4 (51–176)	< 0.001
hsTnT (pg/mL)*	18 (9–42)	13 (7–25)	52.5 (25.5–118)	< 0.001
Art PO2 (mmHg)*	8.8 (7.1–10.7)	9 (7.3–10.8)	8.2 (6.2–10.13)	0.007
Art HCO3^−^ (mmol/L)*	24.7 (22.3–27)	25.1 (23.1–27.5)	23.2 (21.3–25.5)	< 0.001
Art PCO2 (mmHg)*	4.7 (4.1–5.5)	4.8 (4.27–5.6)	4.5 (3.8–5.5)	0.001
ePWV (m/s)*	12 (9.7–14.3)	11 (9.3–13.4)	13.9 (12.5–15.6)	< 0.001
4C Mortality score*	10 (7–13)	9(5–12)	14 (11–15)	< 0.001

Values in parentheses signify SD for continuous variables and percentages for ordinal variables.

*Interquartile range for non-normal continuous variables. Lung disease was defined as at least one of: asthma, chronic obstructive pulmonary disease, interstitial lung disease, obstructive sleep apnoea, home nebuliser/oxygen/non-invasive pressure support. CVD was defined as history of CAD and/or heart failure.

CAD, coronary artery disease; CVD, cardiovascular disease CKD, Chronic Kidney Disease; SBP, Systolic blood pressure; DBP, diastolic blood pressure; PP, pulse pressure; MBP, mean blood pressure; ePWV, estimated pulse wave velocity; WBC, white blood cells CRP, C-reactive protein; hs-Troponin T, high sensitivity Troponin T; Art, arterial; 4C, Coronavirus Clinical Characterisation Consortium) International Severe Acute Respiratory Infection Consortium Clinical Characterisation Protocol.

### Differences of ePWV between patients with COVID-19 and non-COVID controls

**Figure 1:**
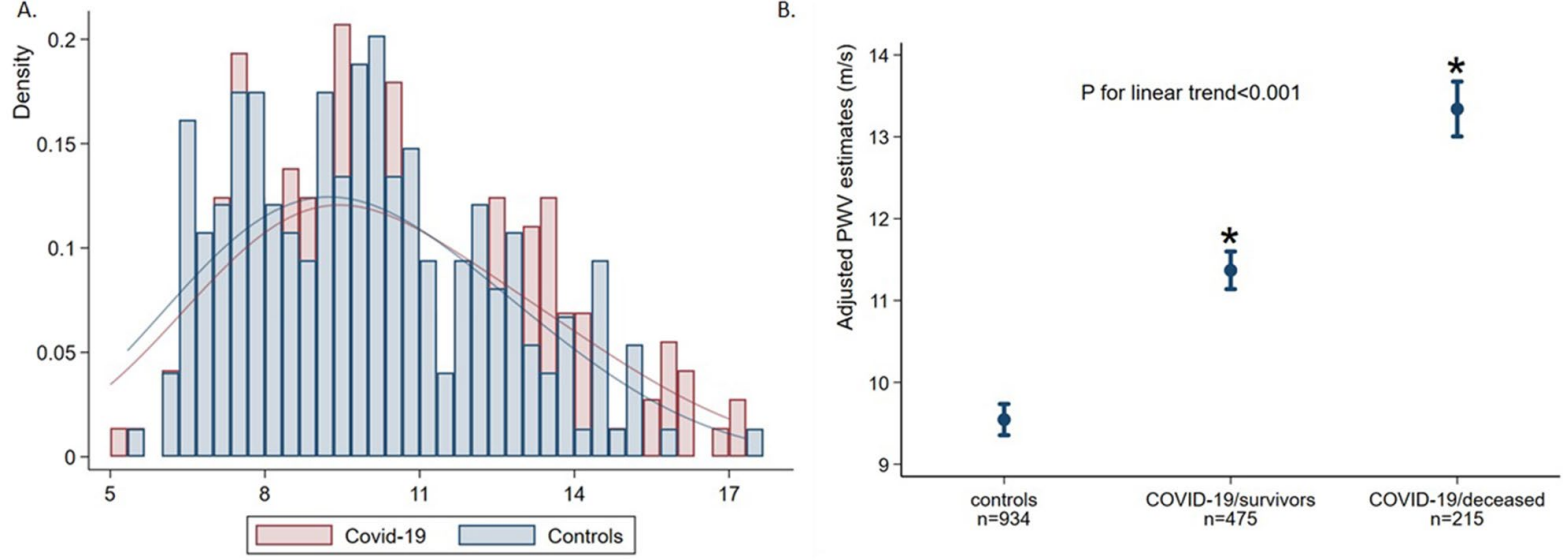
(**A**) Histogram and Kernel Density Estimates of the ePWV in patients with COVID-19 and controls after propensity matching for age, sex, smoking, hypertension, CKD, diabetes mellitus, smoking, history of CVD and hyperlipidemia. (**B**) Difference in ePWV among control subjects without COVID-19, COVID-19 patients who were discharged from hospital and 28-day deceased patients with COVID-19. Estimates of ePWV are adjusted for sex, hypertension, CKD, DM, smoking, history of CVD and hyperlipidemia. Circles represent mean value of ePWV per group and bars the 95% confidence intervals. Asterisks indicate significant (*P* < 0.001) difference from the reference category (i.e., controls from the Athens Vascular Registry). CKD: chronic kidney disease, DM: diabetes mellitus, CVD: cardiovascular disease, including history of coronary artery disease and/or heart failure, PWV: pulse wave velocity.

Propensity score-matching analysis for cardiovascular risk factors (CVRF) yielded a new sample of 233 pairs between patients with COVID-19 and controls. ePWV was significantly higher in COVID-19 patients as compared to their non-COVID-19 counterparts 9.97 m/s (8.44–12.5) vs. 9.56 (7.52–11.5), *p* < 0.001) (Fig. 1A).

When the total cohort of patients with COVID-19 was compared with the total control cohort (n = 934), ePWV progressively increased across the controls, COVID-19 survivors and deceased COVID-19 patients (mean increase in ePWV: 1.89 m/s, 95% CI 1.68–2.1, *P* for linear trend < 0.001) after controlling for CVRFs (Fig. 1B).

A history of CVRFs was similarly associated with higher ePWV in both COVID-19 and control cohorts (Table S4). In the COVID-19, but not in the control cohort (for available parameters), ePWV weakly correlated with laboratory markers of disease severity including C-reactive protein (CRP), white blood cells (WBC), decreased lymphocyte count and high-sensitive Troponin-T (hsTnT) (Table S4).

In the Athens Vascular Registry, we found a good correlation (Pearson’s r = 0.641, *P* < 0.001) between cfPWV and ePWV (Figure S2A-B). A similar association was observed between ePWV and cfPWV in the acute inflammation cohort (Figure S3A-B).

### Classification and reclassification value of ePWV for 28-day mortality

**Figure 2:**
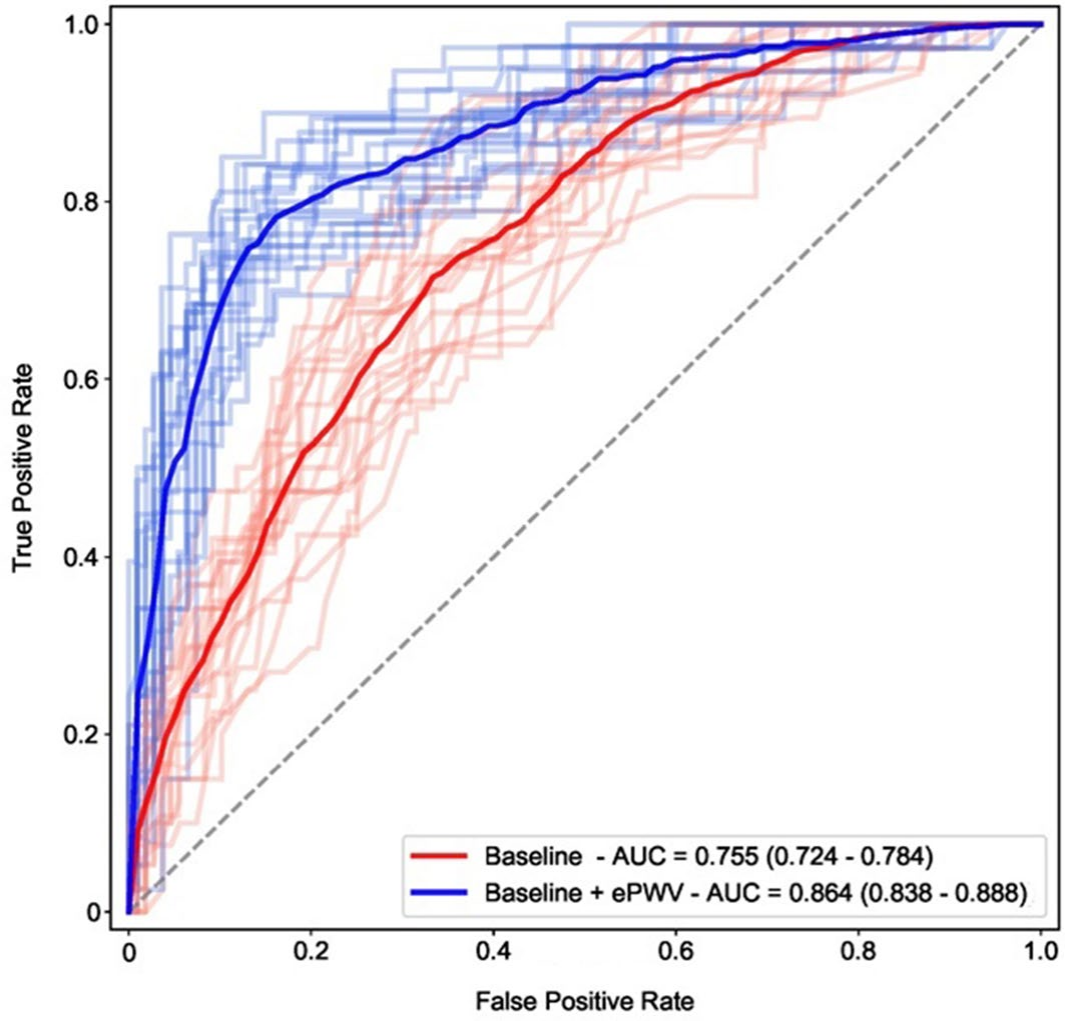
Receiver Operating Characteristic (ROC) curves and corresponding areas under the ROC curve for ePWV on top of the baseline model with respect to 28-day death. Areas under the curve were derived from an appropriate test set (20% of the total sample) after 1,000 bootstrap replicates and training of the boost gradient algorithm on the remaining 80% of the population (training set). Baseline model included age, sex, history of hypertension, DM, CKD, CVD, lung disease and active cancer. To enhance visual clarity a limited number of bootstrapped ROC curves are provided in pale colors as opposed to intense blue and red average estimates. AUC: area under the curve, CKD: chronic kidney disease, DM: diabetes mellitus, CVD: cardiovascular disease including history of coronary artery disease and/or heart failure, ePWV: estimated pulse wave velocity.

By randomly splitting our cohort of patients with COVID-19 (n = 737) into training (80%) and test (20%) sets and replicating our results into 1,000 bootstrapped samples, ePWV significantly improved all classification measures for 28-day death beyond a clinical model with established predictors of adverse prognosis in this disease^4^ (Table 2). Diagnostic accuracy of the reference model increased from 76.4% to 84.5% (*p* < 0.001) while Area under the Curve (AUC) improved from 0.755, (0.724 -0.784) to 0.864, (0.838–0.888, *p* < 0.001) (Fig. 2). Remarkably, ePWV provided superior discrimination for 28-day mortality in patients with COVID-19 as compared to its constituent variables, age and mean blood pressure (MBP) (diagnostic accuracy: 85.1% versus 77.7% and AUC: 0.872 (0.847–0.896) versus 0.788 (0.759–0.817) for ePWV and joint age and MBP respectively, *P* < 0.001 for both) (Table 2).

**Table 2:** Improvement in metrics of classification for 28-day death in COVID-19 after addition of ePWV to a core model of clinical prognostic markers and readily available blood pressure markers.

	Accuracy	Recall (Sensitivity)	Precision (PPV)	AUC	ΔAUC
**28 day mortality (N = 737)**					
Core model*	76.4% (74.3–78.4)	30.0% (23.5–36.8)	54.6% (47.8–61.5)	0.755 (0.724–0.784)	0.109 (0.083–0.135)
Core model + ePWV	84.5% (82.4–86.5)	61.0% (54.3–66.7)	73.1% (67.7–78.2)	0.864 (0.838–0.888)	
Core model + SBP < 120**	77.0% (74.3–79.1)	35.3% (29.0–43.2)	55.6% (48.9–63.0)	0.781 (0.754–0.811)	0.058 (0.065–0.116)
Core model + SBP < 120 + ePWV	84.5% (82.4–86.5)	61.9% (55.6–67.7)	73.3% (68.1–78.8)	0.874 (0.849- 0.897)	
Core model + PP > 60**	77.0% (75.0–79.1)	34.3% (28.2–41.0)	57.1% (50.0–63.6)	0.768 (0.737–0.800)	0.096 (0.072–0.122)
+ Core model PP > 60 + ePWV	84.5% (82.4–86.5)	61.1% (54.6–67.5)	73.1% (68.0–78.4)	0.864 (0.839–0.890)	
Core model + MBP**	77.7% (75.7–79.7)	43.6% (36.8–50.0)	56.7% (51.4–62.5)	0.788 (0.759–0.817)	0.083 (0.058–0.109)
Core model + MBP + ePWV	85.1% (83.1–87.2)	64.9% (59.0–70.6)	73.7% (68.4–78.6)	0.872 (0.847–0.896)	

The core model included age, sex, diabetes mellitus, hypertension, history of cardiovascular disease, lung disease, chronic kidney disease and active cancer.

Core model additionally adjusted for MBP and MBP + ePWV aims to prove the additive prognostic value of ePWV over its constituent variables.

CVD was defined as history of coronary artery disease and/or heart failure.

Results based on 1,000 bootstrap replicates and derived from the test set only (20% of the total sample) after training of the boost gradient algorithm in 80% of the population (random split to training and test set).

Missing values for exposure variables were imputed; thus, all patients were used for classification purposes.

All comparisons to the core model (*) or the core model plus PP > 60 or SPB < 120 or MBP (**) were significant by the non-parametric Mann–Whitney test.

PPV, positive predictive value; AUC, area under the curve; ePWV, estimated pulse wave velocity; SBP, systolic blood pressure; PP, pulse pressure; MBP, mean blood pressure.

Direction of the results for 28-day mortality did not change when non-imputed data for exposure variables were used (Table S5).

Of importance, addition of ePWV improved the classification indices for 28-day death in comparison to an augmented clinical model which included both established risk factors and other readily available BP measures with prognostic value^12^ [i.e., pulse pressure (PP) > 60 mmHg or systolic blood pressure (SBP) < 120 mmHg or SBP > 140 mmHg] (*p* < 0.01 for all comparisons, Table 2 and Table S5). Similar findings were observed in an augmented model with hsTnT (n = 311) (diagnostic accuracy: 88.9% versus 90.5%. *p* < 0.001 and AUC: 0.925, 0.889 -0.952 versus 0.928, 0.894–0.958, *p* = 0.015).

In 590 patients with available information to calculate the 4C Mortality score, ePWV increased the odds of 28-day death by 14% independently of the 4C Mortality score (OR = 1.14 per 1 m/s increase, 95% CI 1.04–1.25, *P* = 0.005). ePWV also conferred additive discrimination (AUC: 0.817, 95% CI 0.780–0.850 versus 0.779, 95% CI 0.743–0.818, *P* < 0.001) and reclassification value (NRI = 0.381, *P* < 0.001) beyond the 4C Mortality score (Table 3). Similarly, ePWV significantly improved the discriminative and reclassification value over the Charlson Comorbidity index (Table 3).

**Table 3:** Additive calibration, discrimination, and reclassification value of ePWV on 4C mortality score for predicting 28-day mortality (n = 626).

	Discrimination								Reclassification			
	AIC	Likelihood ratio chi-squared	*P* value	AUC (95% CI)	ΔAUC (95% CI)	*P* value	IDI (95% CI)	*P* value	Event subjects (%)	Non-event subjects (%)	Overall NRI (95% CI)	*P* value
4C mortality score	524.2	8.96	0.003	0.779 (0.743–0.818)	0.038 (0.028–0.054)	< 0.001	1.2 (0.1–3.6)	0.006	8.7	29.4	0.381 (0.144–0.568)	< 0.001
ePWV	517.2			0.817 (0.780–0.850)								
Charlson comorbidity index	727.1	77.5	< 0.001	0.692 (0.649–0.728)	0.077 (0.058–0.112)	< 0.001	4.5 (1.5–8.5)	< 0.001	17	37.8	0.548 (0.015–0.85)	< 0.001
ePWV	651.5			0.769 (0.732–0.801)								

95% CI are derived from bootstrapping with 1,000 replicates.

4C, Coronavirus Clinical Characterisation Consortium; ePWV, estimated pulse wave velocity; AIC, Akaike information criterion; AUC, area under the curve; NRI, net reclassification index; ΔAUC, difference in AUC; IDI, integrative discrimination index; NRI, net reclassification improvement.

### Sensitivity analyses for the additive classification value of ePWV for 28-day mortality

In 152 patients with obesity characterisation, ePWV improved the classification of established clinical predictors and BMI for 28-day mortality (Table S6). Respectively, ePWV retained its incremental classification value beyond comorbidities when treatment with angiotensin-converting enzyme inhibitor (ACEi) / angiotensin-II receptor blocker (ARB) or history of heart failure were also considered (Table S6). When we further adjusted our baseline model of clinical predictors for the origin of each cohort to account for temporal, regional, and inter-patient differences, the classification ability of ePWV was not diluted (Table S6).

### Clinical cut-offs for ePWV

Aiming to explore the clinical applicability of ePWV for risk stratification in COVID-19 patients, we sought to identify its optimal cut-off points for the prediction of mortality. We found that ePWV value above 13.0 m/s was able to optimally discriminate patients with COVID-19 at high risk for 28-day death (Figure S4). When ePWV was dichotomized according to this cut-off value, increased levels conferred additive prognostic value over the core model (Table S7).

## Discussion

The novel findings of this study are that: ePWV, a readily available estimate of arterial stiffness, is increased in patients hospitalized with COVID-19 versus matched non-COVID-19 controls and provides incremental predictive value for 28-day all-cause mortality beyond established risk factors of adverse outcome for the disease, clustering of comorbidities as assessed by the Charlson comorbidity index and a well-validated mortality score in COVID-19^13^. ePWV was also superior to other risk factors derived from BP measurement previously shown to be associated with mortality such as increased pulse pressure and low SBP^12^. Our results were also consistent in a series of sensitivity analyses including history of heart failure, origin of cohort, patients’ BMI, SBP > 140 mmHg at admission and usage of ACEi/ARBs. These findings suggest that increased arterial stiffness may serve as a predictor of mortality in COVID-19 infection reflecting a cumulative combination of aging, high-risk cardiovascular profile including HMOD and acute vascular dysfunction.

Arterial stiffening is an aging process of the vascular network which is accelerated mainly by hypertension and renal dysfunction^14–16^ but also by other factors such as smoking, diabetes mellitus (DM) and dyslipidemia^17^. Arterial stiffening adversely affects arterio-ventricular coupling leading to heart failure^18^, intercorrelates with endothelial dysfunction^19^, precedes hypertension^17,20^ and is associated with cognitive performance, dementia^21^ and chronic kidney disease (CKD)^16^. In the general population and in hypertensive patients, increased PWV as a measure of arterial stiffness has been associated with HMOD^6^, adverse cardiovascular events and increased all-cause mortality^7,8^ providing incremental and reclassification value over traditional risk factors and total risk scores^7,8,10,11^. ePWV allows swift, easy and reliable assessment of arterial stiffness as a surrogate of PWV with good agreement between the two methods as well as predictive value for adverse cardiovascular events and mortality^9–11^. Interestingly, we have previously shown that PWV increases during acute inflammation^22,23^. The activation of an exaggerated inflammatory status is typical of severe COVID-19 and is associated with organ damage, endothelial cell disruption and intussusceptive angiogenesis^24^. Accordingly, we observed that ePWV was higher in COVID-19 patients as compared to propensity matched non-COVID-19 subjects without an acute inflammatory state. In support of this observation, measured PWV was increased in a separate study of 20 COVID-19 patients as compared to non-COVID-19 controls^25^. More importantly, we observed a graded increase in PWV across groups, peaking in those who died in 28-days period, implying that there may be COVID-19-related mechanisms affecting the observed association between ePWV and mortality. Interestingly, ePWV was also associated with several parameters commonly affected in the acute COVID-19 infection such as increased WBC count, lower lymphocyte count, CRP and hsTnT^26–29^. A role of over activation of the renin–angiotensin–aldosterone system (RAAS) has been implicated in worsening of the status of hospitalized COVID-19 patients based on observed higher MBP and lower serum potassium as markers of RAAS activation in patients with deteriorating respiratory function^30^. To that end, RAAS activation is well established as a pivotal modulator system of arterial stiffening^31^. Nevertheless, adjustment for treatment with ACEi/ARBs did not attenuate the association of ePWV with mortality, suggesting against a critical impact of RAAS inhibition in the prognostic role of this vascular marker. Prospective studies focused on RAAS inhibition should further clarify this issue.

In accordance with our cross-sectional findings, we found that higher ePWV provided incremental prognostic value for 28-day mortality in hospitalized COVID-19 patients. We also found that ePWV was superior to PP and low SBP, which were previously reported to be associated with increased mortality in COVID-19 patients^12^. Furthermore, given that aging, hypertension, history of CVD and CKD are strongly associated with both arterial stiffening^14,16,20^ and adverse prognosis in COVID-19^2,4,5^, our findings imply that ePWV may act as a cumulative death marker of the disease jointly reflecting the severity of a combination of multiple risk factors including HMOD.

Optimizing risk stratification in acute COVID-19 infection is of utmost importance because it may facilitate treatment decisions and timely application of interventions and continuously emerging novel therapies^4,5^. To that end, we found that a value of ePWV ≥ 13.0 m/s optimal for predicting 28-day mortality, respectively. These values are higher than the recommended PWV > 10 m/s for detection of HMOD^6,32^ in the non-COVID population, which is in accordance with previously demonstrated elevation of PWV in the acute phase response^22^.

Several limitations should be acknowledged. Firstly, we used an estimated surrogate of arterial stiffness and not the actual measured marker, possibly limiting the level of correspondence between variability of ePWV values and the process of arterial stiffening particularly in the setting of acute inflammation. However, in addition to previous evidence indicating good agreement with cfPWV, we internally confirmed their close correlation in a large control non-COVID-19 cohort as well as in an independent group of patients with induced acute inflammation^22^. Further, the use of ePWV instead of measured cfPWV offers important practical advantages in this specific population including lower risk of viral transmission, while at the same time cfPWV measurement requires a learning curve and specialized equipment not available in every hospital. Secondly, external validation of our findings in a third independent population was not performed. Still, classification algorithms implemented in our analysis partially circumvent the issue of external validation as they are randomly trained in different populations from the samples in which predictions are made. In this context, we also applied bootstrap techniques and further increased the stability and internal validity of our estimates. Additionally, the recruited cohorts originated from two distinct centers within Europe. Despite the benefits of a multi-center study, in the COVID-19 era this may introduce bias due to differences in recruitment time and local recommendations because new evidence during the pandemic was rapidly emerging. However, the cohorts had overlapping recruitment periods and similar mortality rates as compared with Europe’s reported mortality rates for this period^33^ of the pandemic as well as by month of recruitment. Importantly, adjustment by cohort origin revealed no change in our results. Finally, since baseline ePWV status before COVID-19 infection was unknown, and we used retrospective controls, whether COVID-19 infection per se increased ePWV or its high levels were due to inherent characteristics of the assessed population cannot be proven.

In conclusion, we found that a readily available measure of arterial stiffness provides incremental prognostic value of in hospital mortality in COVID-19 patients beyond known risk factors of the disease as well as the 4C mortality score, a recently well-validated mortality score in hospitalized COVID-19 patients^13^. These findings suggest that ePWV may serve as a novel marker of poor outcome in COVID-19 reflecting a pre-existing adverse risk profile as well as the infection’s effect on the cardiovascular system. Further research should confirm these results and investigate mediating mechanisms.

## Methods

### Population and follow-up

This was a retrospectively designed, longitudinal cohort study examining two independent COVID-19 cohorts from the UK and Italy, a non-COVID cohort from Athens, Greece and an acute inflammation cohort (Figure S1).

### Newcastle COVID-19 cohort

We recorded data for a total of 471 consecutive adults admitted with COVID-19 to the Royal Victoria Infirmary, Newcastle upon Tyne Hospitals NHS Foundation Trust (NUTH), a large tertiary medical centre containing the regional airborne High Consequence Infectious Diseases unit, between 31st January and 31st May 2020. SARS-CoV-2 infection was confirmed by polymerase chain reaction (PCR) testing of combined nose and throat swabs or sputum samples. Demographic and clinical data were retrospectively collected by electronic medical record review. The outcomes and management of the first 316 of these patients have been published elsewhere^34^. All-cause mortality was recorded for all patients until day 28 from admission. The study was registered as a clinical service evaluation with the Newcastle upon Tyne Hospitals NHS Foundation Trust (reference 10,870) and was exempt from ethical approval and was exempt from the requirement for patient consent as a study of COVID-19 under Regulation 3(4) of the Health Service Control of Patient Information Regulations 2002 by the Department of Health and Social Care^35^. Analysis of anonymized healthcare data was approved by the Caldicott Guardian (reference 7595).

### Pisa COVID-19 cohort

Data from 266 patients hospitalized for SARS-CoV-2 pneumonia at the tertiary care University Hospital of Pisa, Tuscany, Italy, between 4th March and 31st March 2020 were retrospectively collected and reviewed through electronic medical records, as previously described^36^. SARS-CoV-2 infection was confirmed by positive results of PCR testing of a nasopharyngeal swab. Demographic, clinical, laboratory, instrumental, treatment, and outcome data were collected according to standard clinical practice and depending on the patient clinical conditions. All-cause mortality was recorded for all patients until day 28 from admission. Informed consent was obtained from all individual participants or their legal guardians included in the study. All experimental protocols were approved by the institutional ethic committee Comitato Etico Area Vasta Nord Ovest (CEAVNO) and were in accordance with the 1975 Declaration of Helsinki.

### Athens non-COVID-19 cohort

These patients are part of an ongoing prospective cohort with > 1,000 recruited individuals (Athens Vascular Registry), as previously described^37,38^. For the purpose of this study, 934 subjects with available cfPWV, aged 20 to 88 years old, were included. The characteristics of this cohort are described in the Table S1. cfPWV was calculated with a validated non-invasive device (Complior, Artech Medical, France)^38,39^. The Local Ethics Committee of Alexandra General Hospital approved the study’s protocol and was in accordance with 1975 Declaration of Helsinki. Before enrollment, all study participants provided written informed consent regarding cfPWV measurement.

### Acute inflammation non-COVID-19 cohort

A previously published cohort from a clinical trial with induced acute inflammation and available cfPWV measurements was used^22^.

### The 4C (Coronavirus Clinical Characterisation Consortium) Mortality Score

The 4C Mortality score is a risk-stratification score developed and validated^13^ from the International Severe Acute Respiratory and emerging Infections Consortium (ISARIC) World Health Organization Clinical Characterisation Protocol UK study, an ongoing prospective study, performed by the ISARIC-4C in 260 hospitals across UK.

### Calculation of ePWV

Using the equations previously derived from the reference Values for Arterial Stiffness’ Collaboration^9^ and implemented in other studies^10,11,40^, ePWV was calculated using age and MBP.

### Statistical analysis

Agreement between cfPWV and ePWV was assessed graphically by Bland–Altman analysis and by calculating the linear correlation (Pearson’s r). In addition, we implemented a multivariable probit regression model and calculated propensity scores for the conditional probability of classification (COVID-19 versus controls) in 737 patients with COVID-19 and 934 subjects with available cfPWV from the Athens Registry. We used linear regression analysis and calculated marginal means for ePWV after adjustment for CVRF, including age sex, hypertension, CKD, DM, smoking, history of CVD and hyperlipidemia to examine differences among pre-specified categories: subjects without COVID-19, patients with COVID-19 who survived, and deceased patients with COVID-19. History of CVD included both coronary artery disease and/or heart failure.

Next, we used machine-learning approach by applying a supervised boost gradient algorithm to assess the predictive value of baseline hemodynamic variables on the classification of patients with COVID-19 into survivors and deceased. We employed a pre-specified baseline set of variables of interest according to previously published medical literature, including age, sex, DM, hypertension, and history of CVD, lung disease, CKD, and active cancer^4^. The machine-learning algorithm was randomly trained in 80% of available observations (training set) prior to generating classification results in the test set (20% of the population). Subsequently, we used boost predictions (i.e., classification probabilities) and calculated median and 25th to 75th percentile of recall (i.e., sensitivity), precision (i.e., positive predictive value), and the AUCs for baseline (“clinical”) and expanded (“clinical plus hemodynamic”) models from 1,000 bootstrapped samples. We also applied the Youden method combined with bootstrapping to derive the optimal cut-off value for ePWV with respect to the prediction of 28-day mortality. Finally, the additive predictive value of ePWV over the ISARIC 4C score was evaluated by calculating the difference in AUCs (ΔAUC), the integrated discrimination improvement (IDI) and the continuous Net Reclassification Index (NRI)^41^. Statistical analysis was conducted with Python 3.7.9 (Python Software Foundation) and STATA 12.1 software (StataCorp, College Station, Texas USA). Full details of our statistical analysis are available in the Data Supplements.

## Supplementary Information

Supplementary Information.
