# Supplementary material for: Estimated pulse wave velocity improves risk stratification for all-cause mortality in patients with COVID-19
Source: Sci Rep. 2021 Oct 12;11:20239. doi: 10.1038/s41598-021-99050-0 (PMC8511157; doi:10.1038/s41598-021-99050-0)
Supplement: Supplementary file 1 — Supplementary Information. [file 41598_2021_99050_MOESM1_ESM.docx]

**Estimated pulse wave velocity improves risk stratification for all-cause mortality in patients with COVID-19**

**Supplementary Material**

Kimon Stamatelopoulos^1,2^, MD*, Georgios Georgiopoulos^1,3^, MD*, Kenneth F. Baker^4,5^, MRCP, PhD*, Giusy Tiseo^6^, MD^#^, Dimitrios Delialis^1^, MD^#^, Charalampos Lazaridis^2,7^, MD, Greta Barbieri^6^, MD, Stefano Masi^6^, MD, PhD, Nikolaos I. Vlachogiannis^2^, MD, Kateryna Sopova^2,7^, MD, Alessandro Mengozzi^6^, MD, Lorenzo Ghiadoni^6^, MD, Ina Schim van der Loeff^4^, MD, PhD, Aidan T. Hanrath^4^, MD, Bajram Ajdini^1^, MSc, Charalambos Vlachopoulos^8^, MD, Meletios A. Dimopoulos^1^, MD, ^4,7^Christopher J.A. Duncan, MD, PhD*, Marco Falcone^6^, MD, PhD*, Konstantinos Stellos^2,7^, MD* and the Pisa COVID-19 Research Group^†^ and Newcastle COVID-19 Research Group^†^

^1^Department of Clinical Therapeutics, National and Kapodistrian University of Athens Medical School, Athens, Greece.

^2^Biosciences Institute, Vascular Biology and Medicine Theme, Faculty of Medical Sciences, Newcastle University, Newcastle Upon Tyne, UK.

^3^School of Biomedical Engineering and Imaging Sciences, King’s College, London, UK.

^4^Translational and Clinical Research Institute, Newcastle University, Newcastle Upon Tyne, UK.

^5^NIHR Newcastle Biomedical Research Centre, Newcastle University and Newcastle upon Tyne Hospitals NHS Foundation Trust

^6^ Department of Clinical and Experimental Medicine, University of Pisa, Pisa, Italy.

^7^RVI and Freeman Hospitals, Newcastle upon Tyne Hospitals NHS Foundation Trust, Newcastle Upon Tyne, UK

^8^First Department of Cardiology, National and Kapodistrian University of Athens Medical School, Athens, Greece.

* equal contribution, ^#^these authors contributed equally

*Address for Correspondence:*

Kimon Stamatelopoulos, Department of Clinical Therapeutics, National and Kapodistrian University of Athens School of Medicine, Athens, Greece. Email: kstamatel@med.uoa.gr or

Konstantinos Stellos, MD; Biosciences Institute, International Centre for Life, Central Parkway, Newcastle upon Tyne, NE1 3BZ, UK; Email: [konstantinos.stellos@ncl.ac.uk](mailto:konstantinos.stellos@ncl.ac.uk)

**I. Members of the Newcastle and Pisa COVID-19 Research Groups**

**II. Population and follow up**

**III. Statistical analysis**

**IV. Supplementary Tables**

**V. Supplementary Figures**

**I. Members of Newcastle and Pisa COVID-19 Research Groups**

*Members of the Pisa’s COVID-19 Research Group:*

Giusy Tiseo^6^, Greta Barbieri^6^, Stefano Masi^6^, Alessandro Mengozzi^6^, Lorenzo Ghiadoni^6^ & Marco Falcone^6^, Fabio Monzani^6^, Francesco Menichetti^6^, Agostino Virdis^6^, Francesco Forfori^6^, Baldassarri Rubia^9^, Bertini Pietro^9^, Brizzi Giulia^9^, Corradi Francesco^9^, Della Rocca Alessandra^9^, Guarracino Fabio^9^, Malacarne Paolo^9^, Monfroni Marco^9^, Piagnani Chiara^9^, Park Naria^9^, Celi Alessandro^10^, Laura Carrozzi^10^, Cinotti Francesco^10^, Massimo Santini^11^, Cipriano Alessandro^11^, Biancalana Martina^11^, Borselli Matteo^11^, Nencini Elia^11^, Spinelli Stefano^11^, Ruberti Francesca^12^, Forotti Giovanna^12^, Sciuto Maria^12^, Salvatore De Marco^12^, Antognoli Rachele^13^, Calsolario Valeria^13^, Paterni Simone^13^, Colangelo Luciano^14^, Sonato Chiara^14^, Galfo Valentina^14^ and Monica Uliana^15^

^6^ Department of Clinical and Experimental Medicine, University of Pisa, Pisa, Italy

^9^ Department of Anaesthesia and Intensive Care, University Hospital of Pisa

^10^ Department of Cardiothoracic and Vascular Department, University of Pisa, Pisa, Italy

^11^ Department of Emergency Medicine, Azienda Ospedaliera Universitaria Pisana, Pisa, Italy

^12^ Fifth Medical Unit, Azienda Ospedaliera Universitaria Pisana, Pisa, Italy

^13^ Geriatrics Unit, Azienda Ospedaliera Universitaria Pisana, Pisa, Italy

^14^ Infectious Diseases Unit, Azienda Ospedaliera Universitaria Pisana, Pisa, Italy

^15^ Fourth Medical Unit, Azienda Ospedaliera Universitaria Pisana, Pisa, Italy

*Members of the Newcastle upon Tyne NHS Foundation Trust (UK) COVID-19 Research Group:*

Kenneth F. Baker^4,5^, Ina Schim van der Loeff^4^, Aidan T. Hanrath^4^, Christopher J.A. Duncan^4,7^, Su Ann Tee^7^, Richard Capstick^7^, Gabriella Marchitelli^7^, Ang Li^7^, Andrew Barr^7^, Alsafi Eid^7^, Sajeel Ahmed^7^, Dalvir Bajwa^7^ and Omer Mohammed^7^

^4^Translational and Clinical Research Institute, Newcastle University, Newcastle Upon Tyne, UK.

^5^NIHR Newcastle Biomedical Research Centre, Newcastle University and Newcastle upon Tyne Hospitals NHS Foundation Trust.

^7^ RVI and Freeman Hospitals, NUTH Newcastle Upon Tyne, UK

**I. Population and follow up**

***Athens ePWV non-COVID cohort***

A total of 934 non-COVID patients, recruited between 2015-2020, were studied in order to assess the association between cfPWV and ePWV measurements. Patients were recruited from the Unit of Dyslipidemias and Atherosclerosis of the Department of Clinical Therapeutics, Alexandra Hospital of the National and Kapodistrian University of Athens, Athens, Greece. 248 (26.6%) patients had a history of ischemic heart disease, defined as at least one of the following: 1) Patients with history of stable angina or acute coronary syndrome ≥ 6 months prior to enrolment; 2) Patients who underwent percutaneous coronary intervention (PCI) or coronary artery bypass graft (CABG) ≥ 6 months prior to enrolment; 3) Patients with cardiac imaging indicative of IHD (i.e. atheromatous plaque causing ≥ 50% lumen stenosis at coronary arteries), stress echocardiography positive for ischemia, myocardial perfusion single-photon emission computed tomography detecting ischemia, or treadmill test positive for ischemia. The majority of patients had at least one cardiovascular risk factor (857/934 (91.8%) total; 248/248 (100%) in those with IHD; 609/686 (88.8%) in those without IHD). CVRFs included the following: active smoking, pre-existing hypertension, hyperlipidemia, diabetes mellitus and chronic kidney disease (CKD, defined as estimated glomerular filtration rate of <60mL/min/m^2^ with the MDRD formula ^1^). Exclusion criteria included the following: withdrawal of consent, life expectancy <1 year, severe valvular heart disease, myocarditis, end-stage heart failure (New York Heart Association (NYHA) IV or left ventricle ejection fraction < 30% ^2^), already scheduled coronary revascularization at baseline visit, end-stage renal failure (GFR<30ml/min/m^2^), active malignancy, autoimmune disease, and active infection. Medical history was obtained from all patients at baseline.

Two different pulse waves were simultaneously obtained with the patient in a supine position at two sites (right common carotid artery and the right femoral artery) using pressure-sensitive transcutaneous transducers. The distance traveled by the pulse wave was measured over the body surface and calculated by subtracting the carotid (sternal notch from the carotid)-femoral distance as distance/time, as previously described^3,4^.

***Acute inflammation non-COVID-19 cohort***

As we have previously shown in a randomized clinical trial that investigated the acute impact of vaccination on arterial stiffness, acute inflammation may increase cfPWV^5^. We therefore sought to assess and validate the correlation between ePWV and cfPWV in the intervention group of that randomized clinical trial In these subjects cfPWV (Complior, Artech Medical) was measured, at baseline, 8 and 32 hours after *Salmonella typhi* vaccination. The correlation between cfPWV and ePWV at 8 hours was assessed.

**Calculation of ePWV**

For subjects with at least one cardiovascular risk factor (CVRF), systolic blood pressure (SBP) ≥140mmHg or diastolic blood pressure (DBP) ≥90mmHg or history of CVD: ePWV = 9.587–(0.402×age)+[4.560×10^−3^×(age)^2^] – [2.621×10^−5^×(age)^2^×MBP] +(3.176×10^−3^× age×MBP)–(1.832×10^−2^×MBP) [reference population]. For subjects without any CVRF and SBP<140mmHg and DBP<90mmHg, without a history of CVD, ePWV= 4.6 – [0.1×age+0.0018×(age)^2^] + (6×10^-4^×age×MBP)+(2.8×10^-2^×MBP) [normal population]. MBP was calculated as DBP+0.4×(SBP-DBP). Because inflammation is considered a condition that increases PWV^6^, the formula for reference population was used in all COVID-19 patients.

**II. Statistical analysis**

Variables of interest were compared between survivors and patients who deceased with the independent’s samples Student’s T Test or the non-parametric Mann-Whitney test and the chi-squared test for continuous and nominal variables, respectively. Associates of ePWV were identified by using the Spearman’s correlation coefficient. Agreement between cfPWV and ePWV was assessed graphically by Bland-Altman analysis and by calculating the linear correlation (Pearson’s r). We implemented a multivariable probit regression model and calculated propensity scores for the conditional probability of classification (COVID-19 versus controls) in 737 patients with COVID-19 and 937 subjects from the Athens Vascular Registry. Baseline characteristics (age and sex), previous history of IHD, risk factors and co-morbidities (diabetes mellitus, hypertension and CKD) as well as SBP were considered for the calculation of the propensity score for each subject. We employed a 1:1 matching algorithm of the nearest neighbor with no replacement; the caliper was set at 0.05 (equal to 0.2*standard deviation of the propensity score)^7^. Covariate balance between matched patients was assessed by Student’s T-Tests for equality of means, the reduction in the standardized percentage bias and measures of overall covariate balance (i.e., median overall bias and Rubin’s R within the range 0.5 to 2). We used the non-parametric Wilcoxon signed-rank test to compare ePWV between patients with COVID-19 and controls in the final matched sample.

Next, we used machine-learning approach by applying a supervised gradient boosting algorithm to assess the predictive value of baseline hemodynamic variables on the classification of patients with COVID-19 into survivors and deceased. In detail, we implemented a stochastic gradient boosting algorithm with classification decision trees for patients who deceased and survived a) during and b) after hospitalization^8,9^. We employed a pre-specified baseline set of variables of interest according to previously published medical literature, including age, sex, diabetes, hypertension, history of cardiovascular disease, lung disease, chronic kidney disease and active cancer. We used a grid search optimization algorithm to fine-tune gradient boosting parameters, including number of trees, learning rate, maximum depth and maximization features and ultimately to minimize out-of-bag and validation error. The machine-learning algorithm was randomly trained in 80% of available observations (training set) prior to generating classification results in the test set (20% of the population). Up to two-way interactions among predictors were evaluated in boost models. We constructed variable importance plots to rank baseline variables in classifying dead and alive patients with COVID-19. Variable importance plots were derived from changes in prediction accuracy in the out-of-bag sample following sequential removal (by random shuffling values) of variables of interest. The relative importance (importance for each variable was divided by the highest variable importance) was bounded between 0 and 1. Subsequently, we used boost predictions (i.e., classification probabilities) and calculated recall (i.e., sensitivity), precision (i.e., positive predictive value) and the Area Under the Curve (AUCs) for baseline (“clinical”) and expanded (“clinical plus hemodynamic”) models. The additive predictive value of hemodynamic variables on top of clinical characteristics was evaluated by comparing AUCs with non-parametric tests. We performed validation of boost models after bootstrapping with replacement to increase confidence in reported model estimates and calculated the median and 25^th^ and 75^th^ percentile of classification statistics from 1,000 bootstrapped samples. We also applied the Youden method combined to bootstrapping to derive the optimal cut-off ePWV values for prediction of the outcome; then, we repeated our gradient boosting algorithms by implementing ePWV as a dichotomous variable (above and below the cut-off). Finally, we performed logistic regression analysis to test the independent association of ePWV with 28-days mortality after controlling for the ISARIC 4C score; we further evaluated the additive predictive value of ePWV over this established prognostic system (4C Mortality Score) and the Charlson comorbidity Index by calculating i) the improvement in fitting of model predictions to events through likelihood ratio tests and decrease in the Akaike Information Criterion ii) the difference in AUCs (ΔAUC) iii) the integrated discrimination improvement (IDI) and iv ) the continuous Net Reclassification Index (NRI)^10^. The 95% confidence intervals around the mean estimates for ΔΑUC and NRI were constructed by bootstrapping (1,000 replicates) with replacement.

Baseline demographic variables had <1% missing values. For hemodynamic indices with missing observations (~6.3%), we used multiple imputation with the Monte Carlo Markov Chain method and added 20 datasets after 5 additional iterations for the burn-in period prior to applying the gradient boosting algorithm for classification.^11^ Machine learning algorithms were performed on both imputed and non-imputed data as a sensitivity analysis.

A sample size of 650 patients was adequately powered at 0.9 level to detect a clinically significant difference of 0.5m/s difference in ePWV between patients with COVID-19 who survived, and deceased given a standard deviation of 0.5. Measures of tendency and dispersion for ePWV were retrieved from the Athens Vascular registry and type I error was predefined at a=0.05. A priori simulations showed an out-of-sample squared root of the mean squared prediction error of 0.035 at a sample size of N= 600, with 8 candidate predictors and an event fraction of 0.25. Statistical analysis was conducted with Python and STATA 12.1 software (StataCorp, College Station, Texas USA). We deemed statistical significance at P<0.05.

**III. Supplementary Tables**

| **Table S1. Characteristics of patients from the Athens Vascular registry (N=934)** | | |
| --- | --- | --- |
|  |  | **Mean (SD) / N (%)** |
| Age (years) |  | 59.9 (12.7) |
| Sex (male) |  | 527 (56.3) |
| CKD (N) |  | 137 (14.7) |
| Smoking (N) |  | 316 (33.8%) |
| CVD (N) |  | 248 (26.6) |
| Diabetes mellitus (N) |  | 195 (20.9) |
| Hypertension (N) |  | 470 (50.3) |
| Hyperlipidemia (N) |  | 609 (65.3) |
| Cancer (N) |  | N/A |
| Lung disease (N) |  | N/A |
| SBP (mmHg) |  | 130.9 (20.5) |
| DBP (mmHg) |  | 73.2 (10.8) |
| PP (mmHg) |  | 57.7 (16.7) |
| MBP (mmHg) |  | 96.3 (13.1) |
| ePWV (m/sec) |  | 9.7 (2.2) |
| cfPWV (m/sec) |  | 10.1 (2.8) |
| Lung disease was defined as at least one of: asthma, chronic obstructive pulmonary disease, interstitial lung disease, obstructive sleep apnoea, home nebuliser/oxygen/non-invasive pressure support. CVD was defined as history of coronary artery disease and heart failure. CVD: Cardiovascular disease, CKD: Chronic Kidney Disease, SBP: Systolic blood pressure, DBP: diastolic blood pressure, PP: pulse pressure, MBP: mean blood pressure, PWV: pulse wave velocity. P-values are derived from paired samples Student’s T Test or the Wilcoxon Rank Sum Test for continuous variables and the McNemar’s chi squared test for categorical variables. | | |

| **Supplementary Table S2.** 28-days Mortality rates in the two cohorts by month of recruitment | | |
| --- | --- | --- |
| Newcastle cohort | | |
| March 2020 | April 2020 | May 2020 |
| 26.9% | 24.5% | 30% |
| Pisa cohort | | |
| March 2020 | April 2020 | May 2020 |
| 24.6% | 16.7% | N/A |
|  | | |

| **Table S3. Descriptive and comparison of the characteristics of the combined Newcastle and Pisa cohorts of hospitalised patients with COVID-19** | | | | | |
| --- | --- | --- | --- | --- | --- |
|  | N | All | Pisa Cohort | Newcastle cohort | P-Value |
| Age (years) | 737 | 69.2 (17) | 67.6 (15.7) | 70.1 (17.7) | 0.052 |
| Sex (female) | 737 | 309 (41.9) | 86 (32.3) | 223 (47.4) | <0.001 |
| CKD (N) | 735 | 136 (18.5) | 23 (8.7) | 113 (24.1) | <0.001 |
| CAD (N) | 735 | 181 (24.6) | 76 (28.6) | 95 (20.3) | 0.006 |
| Heart failure | 735 | 93 (12.6) | 11 (4.1) | 68 (14.5) | <0.001 |
| Diabetes mellitus (N) | 737 | 175 (23.7) | 51 (19.2) | 124 (26.3) | 0.028 |
| Hypertension (N) | 735 | 311 (42.3) | 122(45.9) | 189 (40.3) | 0.142 |
| Cancer (N) | 737 | 95 (12.9) | 39(14.7) | 56 (11.9) | 0.281 |
| Lung disease (N) | 737 | 169 (22.93) | 49 (18.4) | 120 (25.5) | 0.029 |
| Smoking (N) | 652 | 28 (4.29) | 12 (5.0) | 16 (3.9) | 0.486 |
| Hyperlipidemia (N) | 737 | 44 (6.0) | 44 (16.5) | 0 (0.00) | <0.001 |
| SBP (mmHg) | 690 | 129 (22.5) | 131 (20.9) | 127 (23.3) | 0.028 |
| DBP (mmHg) | 690 | 73.7 (14) | 74.8 (13.5) | 73.1 (14.3) | 0.142 |
| PP (mmHg) | 690 | 54.9 (19.2) | 56.4 (15.5) | 54.1 (20.8) | 0.134 |
| 28 days death (N) | 737 | 184 (25.0) | 64 (24.1) | 120 (25.5) | 0.669 |
| *Days of hospitalization | 724 | 10 (5-19) | 15 (7.5-24) | 8.5 (4-16) | <0.001 |
| *WBC (10^3^/ml) | 719 | 7.12 (5.3-9.4) | 6.59 (5.0-9.1) | 7.45 (5.5-9.7) | 0.025 |
| *Lympocytes (10^3^/ml) | 717 | 9.40 (6.40-13.30) | 9.50 (6.40-13.00) | 9.30 (6.55-13.75) | 0.613 |
| *CRP (mg/L) | 701 | 68(28-135) | 67.3(30.8-140.9) | 68(27-134) | 0.406 |
| *hsTnT (pg/mL) | 311 | 18 (9-42) | 17 (9-40) | 18 (9-48) | 0.499 |
| *PO2 (mmHg) | 434 | 8.8 (7.1-10.7) | 9.2 (7.6-11.3) | 8.4 (6.5-10.1) | <0.001 |
| *HCO3^-^ (mmol/L) | 457 | 24.6(22-27) | 24.2(21.9-27) | 24.7 (22.3-27) | 0.444 |
| *PCO2 (mmHg) | 508 | 4.7 (4.1-5.5) | 4.53 (4.0-5.1) | 4.9 (4.2-5.8) | <0.001 |
| *ePWV (m/sec) | 690 | 12 (9.7-14.3) | 11.6 (9.6-13.8) | 12.2 (9.7-14.5) | 0.134 |
| *4C Mortality score | 590 | 10(7-13) | 9(5-13) | 10(8-13) | <0.001 |
| Values in brackets signify SD for continuous variables, *IQR for non-normal continuous variables and percentages for ordinal variables. Lung disease was defined as at least one of: asthma, chronic obstructive pulmonary disease, interstitial lung disease, obstructive sleep apnoea, home nebuliser/oxygen/non-invasive pressure support. CVD was defined as history of CAD and/or heart failure. CAD: coronary artery disease, CVD: cardiovascular disease, CKD: Chronic Kidney Disease, SBP: Systolic blood pressure, DBP: diastolic blood pressure, PP: pulse pressure, MBP: mean blood pressure, ePWV: estimated pulse wave velocity, WBC: white blood cells CRP: C-reactive protein, hs-Troponin T: high sensitivity Troponin T, Art: arterial | | | | | |

| **Table S4. Correlation of ePWV with comorbidities and known risk factors for adverse outcome in COVID-19 patients and non-COVID-19 subjects** | | | | |
| --- | --- | --- | --- | --- |
|  | COVID-19 (N=737) | | Non-COVID (N=934) | |
|  | r | p | r | p |
| **Male sex** | **0.139** | **<0.001** | **0.204** | **<0.001** |
| **Hypertension** | **0.305** | **<0.001** | **0.471** | **<0.001** |
| Cancer | 0.062 | 0.102 | N/A | N/A |
| **Lung disease** | **0.113** | **0.003** | N/A | N/A |
| **CVD** | **0.234** | **<0.001** | **0.290** | **<0.001** |
| **Diabetes mellitus** | **0.096** | **0.012** | **0.217** | **<0.001** |
| **CKD** | **0.209** | **<0.001** | **0.407** | **<0.001** |
| CRP | **0.321** | **<0.001** | 0.003 | 0.925 |
| **hsTnT (N=311)** | **0.578** | **<0.001** | N/A | N/A |
| **Lymphocyte count** | **-0.118** | **0.002** | N/A | N/A |
| **WBC count** | **0.118** | **0.002** | -0.019 | 0.619 |
| PaO2 | -0.088 | 0.077 | N/A | N/A |
| PaC02 | 0.073 | 0.11 | N/A | N/A |
| **HCO3-** | **-0.145** | **0.018** | N/A | N/A |
| r: Spearman’s rank correlation coefficient. P-value derived from Spearman’s correlation coefficient. Lung disease was defined as at least one of: asthma, chronic obstructive pulmonary disease, interstitial lung disease, obstructive sleep apnoea, home nebuliser/oxygen/non-invasive pressure support. CVD was defined as history of coronary artery disease and/or heart failure. CKD: Chronic Kidney Disease, CVD: cardiovascular disease, ePWV: estimated pulse wave velocity, CRP: C-reactive protein, hsTnT: high sensitive troponin T, WBC: white blood cells , Art: arterial, N/A not available | | | | |

| **Table S5. Improvement in metrics of classification for death in COVID-19 after addition of ePWV for mortality on a core model of clinical prognostic markers or clinical prognostic markers plus blood pressure measurements by using list wise deletion of missing observations** | | | | |
| --- | --- | --- | --- | --- |
|  | Accuracy | Recall (Sensitivity) | Precision  (PPV) | AUC |
| **28-days mortality (N=690)** | | | | |
| Baseline model* | 82.6 %  (80.4 -84.8) | 60.0 %  (53.3 - 65.8) | 68.8%  (63.6-73.9) | 0.851  (0.825 -0.878) |
| Baseline model+ePWV | 87 %  (84.8 – 89.1) | 70.3 %  (63.9-75.7) | 77.1 %  (71.9-82.1) | 0.891  (0.865-0.913) |
| Baseline model+SBP<120** | 84.1 %  (81.9-86.2) | 62.9%  (56.8-68.8) | 70.8 %  (65.6-76.2) | 0.869  (0.841-0.893) |
| Baseline model+SBP<120+ePWV | 87 %  (84.8-89.1) | 70.3%  (63.6-75.7) | 76.7%  (72.2-81.5) | 0.893  (0.868-0.915) |
| Baseline model+PP>60** | 84.1 %  (81.9-86.2) | 62.2%  (55.9-67.7) | 71 %  (65.4-75.9) | 0.861  (0.835-0.888) |
| Baseline model+PP>60+ePWV | 86.7 %  (85.6-89.1) | 70.3%  (64.5-75.8) | 77.4%  (72.4-82.1) | 0.894  (0.870-0.916) |
| PPV: positive predictive value, AUC: area under the curve, ePWV: estimated pulse wave velocity, SBP: systolic blood pressure, PP: pulse pressure  Cut-offs for ePWV were derived by maximizing Youden’s index in 1,000 bootstrap replicates  The reference model included age, sex, diabetes mellitus, hypertension, history of cardiovascular disease, lung disease, chronic kidney disease and active cancer.  CVD was defined as history of coronary artery disease and/or heart failure.  Results based on 1,000 bootstrap replicates and derived from the test set only (20% of the total sample) after training of the boost gradient algorithm in 80% of the population (random split to training and test set)  Missing values for exposure variables were imputed; thus, all patients were used for classification purposes.  All comparisons to the reference model (*) or were significant by the non-parametric Mann-Whitney test | | | | |

| **Table S6. Sensitivity analyses for improvement in metrics of classification for 28-days death in COVID-19 after addition of ePWV to a core model of clinical prognostic markers and readily available blood pressure markers** | | | | |
| --- | --- | --- | --- | --- |
|  | **Accuracy** | **Recall (Sensitivity)** | **Precision**  **(PPV)** | **AUC** |
| **28-day mortality** | | | | |
| Core model +BMI (n=152) | 87.1 %  (83.9 -90.3) | 57.1 %  (42.9-71.4) | 77.8 %  (66.7-100.0) | 0.857  (0.785- 0.919) |
| Core model+BMI and ePWV | 90.3 %  (87.1-93.6) | 66.7 %  (50.0-83.3) | 85.7 %  (75.0-100.0) | 0.897  (0.823-0.954 |
| Core model +ACEi/ARBs (n=726) | 76.0 %  (74.0-78.1) | 25.0 %  (18.9-31.6) | 55.6 %  (47.4-64.7) | 0.753  (0.721-0.785) |
| Core model +ACEi/ARBs and ePWV | 80.8 %  (78.8-82.2) | 43.2 %  (37.1-50.0) | 68.2 %  (61.9-73.9) | 0.830  (0.805-0.858) |
| Core model +HF (n=737) | 76.9%  (74.8-78.9) | 25 %  (19.4-30.8) | 59.1 %  (50-68.2) | 0.753  (0.721-0.786) |
| + Core model HF+ePWV | 81 %  (78.9-83) | 44.7 %  (38.2-51.4) | 67.8 %  (61.1-73.7) | 0.834  (0.806-0.860) |
| Age + SBP>140 (n=737) | 77.0 %  (74.3-78.4) | 25.0 %  (18.9-31.7) | 57.1 %  (50.0-66.7) | 0.797  (0.742-0.797) |
| Age + SBP>140 + ePWV | 81.1 %  (79.1-83.1) | 47.6 %  (40.5-54.1) | 68.0 %  (61.9-73.3) | 0.849  (0.826-0.873) |
| Core model + cohort (n=737) | 77.0 %  (75.0-78.4) | 25.0 %  (18.9-31.6) | 58.8 %  (50.0-66.7) | 0.756  (0.725-0.784) |
| Core model + cohort+ ePWV | 81.1 %  (79.1-82.4) | 44.1 %  (37.8-50.0) | 68.2 %  (62.0-75.0) | 0.836  (0.811-0.862) |
| PPV: positive predictive value, AUC: area under the curve, ePWV: estimated pulse wave velocity, SBP: systolic blood pressure, PP: pulse pressure, MBP: mean blood pressure, ACEi: angiotensin-converting enzyme inhibitor, ARB: angiotensin-II receptor blocker, HF: heart failure.  The reference model included age, sex, diabetes mellitus, hypertension, history of cardiovascular disease, lung disease, chronic kidney disease and active cancer.  CVD was defined as history of coronary artery disease and/or heart failure.  Results based on 1,000 bootstrap replicates and derived from the test set only (20% of the total sample) after training of the boost gradient algorithm in 80% of the population (random split to training and test set)  Missing values for exposure variables were imputed; thus, all patients were used for classification purposes.  All comparisons to the core model plus BMI or ACEi/ARBs were significant when adding ePWV by the non-parametric Mann-Whitney test | | | | |

| **Table S7. Improvement in metrics of classification for death in COVID-19 after addition of dichotomous ePWV by optimal cutoff value for mortality on a core model of clinical prognostic markers and readily available blood pressure markers** | | | | |
| --- | --- | --- | --- | --- |
|  | Accuracy | Recall (Sensitivity) | Precision  (PPV) | AUC |
| **28-days mortality (N=737)** | | | | |
| Baseline model* | 76.4 %  (74.3 -78.4) | 30.0 %  (23.5 - 36.8) | 54.6%  (0.478-0.615) | 0.755  (0.724 -0.784) |
| Baseline model+ePWV≥13m/sec | 77.7 %  (75.7 – 79.7) | 43.2 %  (36.8-50.3) | 57.6 %  (51.7-64.0) | 0.802  (0.771-0.827) |
| Baseline model+SBP<120** | 77.0 %  (74.3-79.1) | 35.3 %  (29.0-43.2) | 55.6 %  (48.9-63.0) | 0.781  (0.754-0.811) |
| Baseline model+SBP<120  +ePWV≥13m/sec | 79.7 %  (77.7-81.8) | 48.6 %  (42.1-55.3) | 62.5 %  (56.5-68.2) | 0.830  (0.802-0.852) |
| Baseline model+PP>60** | 77.0 %  (75.0-79.1) | 34.3 %  (28.2-41.0) | 57.1 %  (50.0-63.6) | 0.768  (0.737-0.800) |
| Baseline model+PP>60  +ePWV≥13m/sec | 79.1 %  (77.0-81.1) | 47.2 %  (40.0-53.7) | 60.9 %  (55.2-66.7) | 0.813  (0.785-0.840) |
| PPV: positive predictive value, AUC: area under the curve, ePWV: estimated pulse wave velocity, SBP: systolic blood pressure, ePWV: estimated pulse wave velocity, PP: pulse pressure  Cut-offs for ePWV were derived by maximizing Youden’s index in 1,000 bootstrap replicates  The reference model included age, sex, diabetes mellitus, hypertension, history of cardiovascular disease, lung disease, chronic kidney disease and active cancer.  CVD was defined as history of coronary artery disease and/or heart failure.  Results based on 1,000 bootstrap replicates and derived from the test set only (20% of the total sample) after training of the boost gradient algorithm in 80% of the population (random split to training and test set)  Missing values for exposure variables were imputed; thus, all patients were used for classification purposes.  All comparisons to the reference model (*) or were significant by the non-parametric Mann-Whitney test | | | | |

**IV. Supplementary Figures**


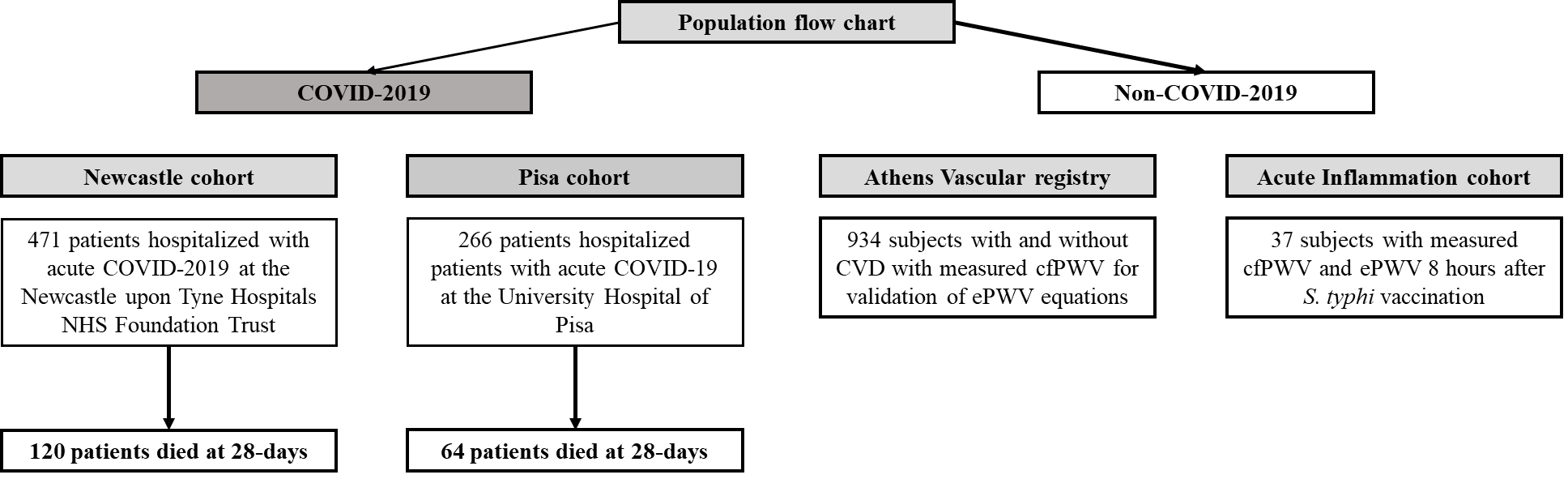


**Figure S1.** Flow chart of the populations used in this study.


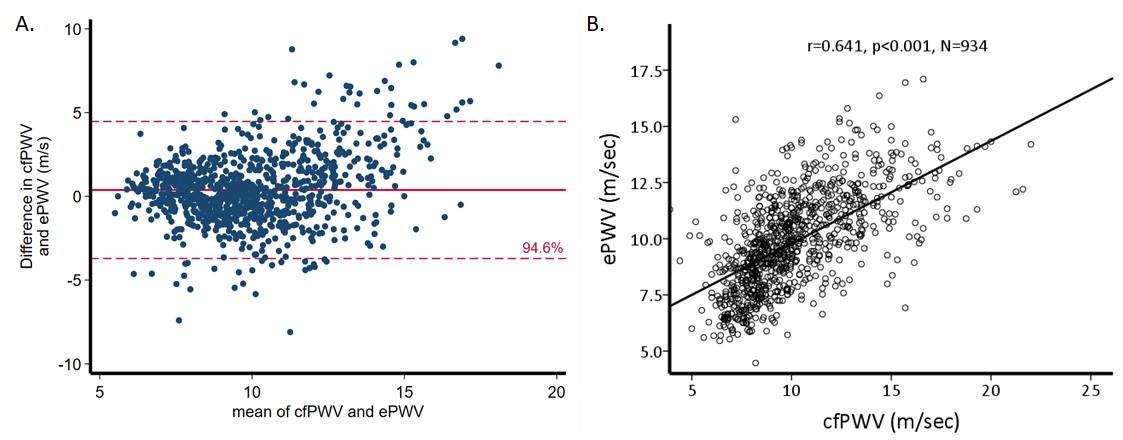


**Figure S2. Bland-Altman plot (A) and linear correlation (B) for measurements of cfPWV and ePWV in Athens Vascular Registry.** The percentage indicate the percentage of paired measurements that fall within the 2 SDs of the mean difference of all pairs. A positive bias indicates higher cfPWV values as compared to ePWV across increasing values. Pearson’s correlation coefficient alongside corresponding P-value are provided for linear correlation. cfPWV: carotid-femoral pulse wave velocity, ePWV: estimated PWV, SD: standard deviation


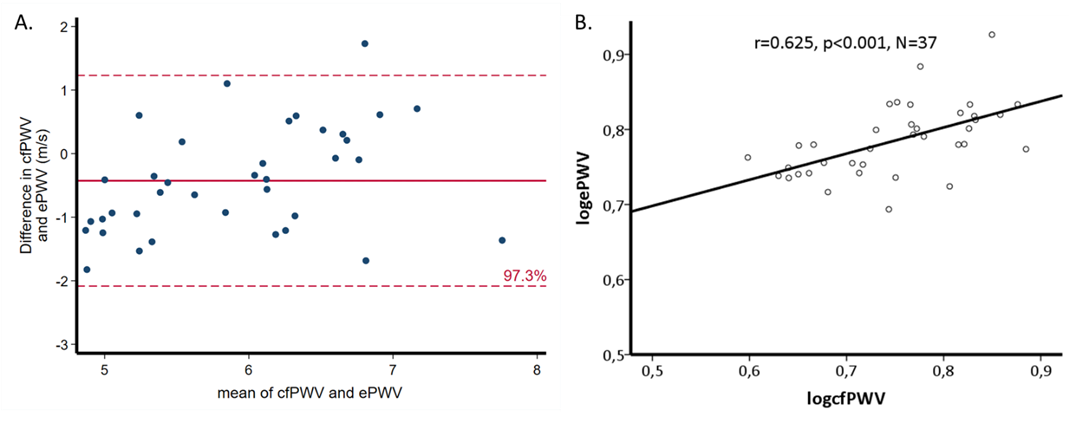


**Figure S3. Bland-Altman plot (A) and linear correlation (B) for measurements of cfPWV and ePWV in the acute inflammation cohort.** The percentage indicate the percentage of paired measurements that fall within the 2 SDs of the mean difference of all pairs. A positive bias indicates higher cfPWV values as compared to ePWV across increasing values. Pearson’s correlation coefficient alongside corresponding P-value are provided for linear correlation. cfPWV: carotid-femoral pulse wave velocity, ePWV: estimated PWV, SD: standard deviation


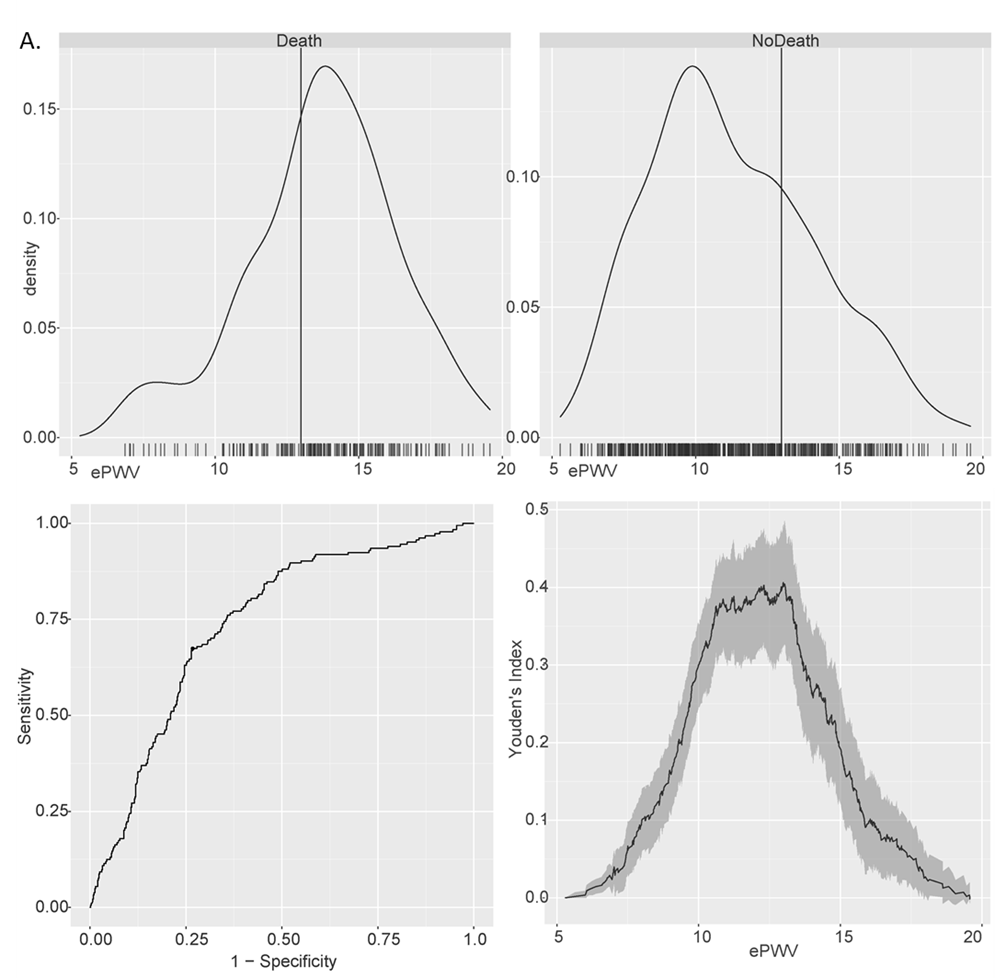


**Figure S4.** Derivation of cut-off points for ePWV and 28-days mortality by maximizing the Youden’s index in 1,000 bootstrap replicates of our sample (n=737). ePWV: estimated pulse wave velocity.

**References**

1 Levey, A. S. *et al.* A new equation to estimate glomerular filtration rate. *Annals of internal medicine* **150**, 604-612, doi:10.7326/0003-4819-150-9-200905050-00006 (2009).

2 Ponikowski, P. *et al.* 2016 ESC Guidelines for the diagnosis and treatment of acute and chronic heart failure: The Task Force for the diagnosis and treatment of acute and chronic heart failure of the European Society of Cardiology (ESC)Developed with the special contribution of the Heart Failure Association (HFA) of the ESC. *Eur Heart J* **37**, 2129-2200, doi:10.1093/eurheartj/ehw128 (2016).

3 Stamatelopoulos, K. *et al.* Amyloid-beta (1-40) and the risk of death from cardiovascular causes in patients with coronary heart disease. *J Am Coll Cardiol* **65**, 904-916, doi:10.1016/j.jacc.2014.12.035 (2015).

4 Stamatelopoulos, K. *et al.* Physical activity is associated with lower arterial stiffness in normal-weight postmenopausal women. *J Clin Hypertens (Greenwich)* **22**, 1682-1690, doi:10.1111/jch.13954 (2020).

5 Vlachopoulos, C. *et al.* Acute systemic inflammation increases arterial stiffness and decreases wave reflections in healthy individuals. *Circulation* **112**, 2193-2200, doi:10.1161/circulationaha.105.535435 (2005).

6 Pietri, P. *et al.* Relationship between low-grade inflammation and arterial stiffness in patients with essential hypertension. *Journal of hypertension* **24**, 2231-2238, doi:10.1097/01.hjh.0000249701.49854.21 (2006).

7 Abadie, A., Drukker, D., Herr, J. L. & Imbens, G. W. Implementing Matching Estimators for Average Treatment Effects in Stata. *The Stata Journal* **4**, 290-311, doi:10.1177/1536867x0400400307 (2004).

8 Galar, M., Fernandez, A., Barrenechea, E., Bustince, H. & Herrera, F. A Review on Ensembles for the Class Imbalance Problem: Bagging-, Boosting-, and Hybrid-Based Approaches. *IEEE Transactions on Systems, Man, and Cybernetics, Part C (Applications and Reviews)* **42**, 463-484, doi:10.1109/TSMCC.2011.2161285 (2012).

9 Stollhoff, R., Sauerbrei, W. & Schumacher, M. An experimental evaluation of boosting methods for classification. *Methods Inf Med* **49**, 219-229, doi:10.3414/me0543 (2010).

10 Pencina, M. J., D'Agostino Sr, R. B. & Steyerberg, E. W. Extensions of net reclassification improvement calculations to measure usefulness of new biomarkers. *Statistics in Medicine* **30**, 11-21, doi:<https://doi.org/10.1002/sim.4085> (2011).

11 Stamatelopoulos, K. *et al.* Amyloid-β (1-40) and Mortality in Patients With Non-ST-Segment Elevation Acute Coronary Syndrome: A Cohort Study. *Annals of internal medicine* **168**, 855-865, doi:10.7326/m17-1540 (2018).
